# Crystal structures of three 4-substituted-2,2′-bipyridines synthesized by Sonogashira and Suzuki–Miyaura cross-coupling reactions

**DOI:** 10.1107/S2056989017004662

**Published:** 2017-03-31

**Authors:** Thuy Luong Thi Thu, Ngan Nguyen Bich, Hien Nguyen, Luc Van Meervelt

**Affiliations:** a Department of Chemistry, Hanoi National University of Education, 136 Xuan Thuy, Cau Giay, Hanoi, Vietnam; b Department of Chemistry, KU Leuven, Biomolecular Architecture, Celestijnenlaan 200F, Leuven (Heverlee), B-3001, Belgium

**Keywords:** crystal structure, dye-sensitized solar cells, 2,2′-bi­pyridine, palladium-catalyzed, Sonogashira cross-coupling, Suzuki–Miyaura cross-coupling

## Abstract

Facile synthetic routes for three 4-substituted 2,2′-bi­pyridine derivatives, 4-[2-(4-methyl­phenyl)­ethyn­yl]-2,2′-bi­pyridine, C_19_H_14_N_2_, (I), 4-[2-(pyridin-3-yl)ethyn­yl]-2,2′-bi­pyridine, C_17_H_11_N_3_, (II), and 4-(indol-4-yl)-2,2′-bi­pyridine, C_18_H_13_N_3_, (III), *via* Sonogashira and Suzuki–Miyaura cross-coupling reactions, respect­ively, are described. As indicated by X-ray analysis, the 2,2′-bi­pyridine core, the ethyl­ene linkage and the substituents of (I) and (II) are almost planar [dihedral angles between the two ring systems: 8.98 (5) and 9.90 (6)° for the two mol­ecules of (I) in the asymmetric unit and 2.66 (14)° for (II)], allowing π-conjugation. On the contrary, in (III), the indole substituent ring is rotated significantly out of the bi­pyridine plane [dihedral angle = 55.82 (3)°], due to steric hindrance. The crystal packings of (I) and (II) are dominated by π–π inter­actions, resulting in layers of mol­ecules parallel to (30-2) in (I) and columns of mol­ecules along the *a* axis in (II). The packing of (III) exhibits zigzag chains of mol­ecules along the *c* axis inter­acting through N—H⋯N hydrogen bonds and π–π inter­actions. The contributions of unknown disordered solvent mol­ecules to the diffraction intensities in (II) were removed with the SQUEEZE [Spek (2015 ▸). *Acta Cryst.* C**71**, 9–18] algorithm of *PLATON*. The given chemical formula and other crystal data do not take into account these solvent mol­ecules.

## Abstract 2

In the crystal structures of three 4-substituted-2,2′-bi­pyridines prepared using facile synthetic procedures, two novel 4-alkynyl-2,2-bi­pyridines *via* the Sonogashira cross-coupling reaction and one 4-aryl-2,2′-bi­pyridine *via* the Suzuki–Miyaura cross-coupling reaction, the planar 4-alkynyl-substituted derivatives are in contrast to the non-planar 4-aryl derivative.

## Chemical context   

The bidentate ligand 2,2′-bi­pyridine (Bpy) is one of the most studied chelate systems and has found applications in various fields, including catalysis (Kitanosono *et al.*, 2015 ▸; Song *et al.*, 2015 ▸), chemosensors for metal ions (Al Abdel Hamid *et al.*, 2011 ▸), electroluminescent devices (Li *et al.*, 2000 ▸), and mol­ecular shuttles (Lewis *et al.*, 2016 ▸). In particular, as a result of their unique photophysical characteristics, 2,2′-bi­pyridine derivatives are used in the synthesis of photosensitizers (Grätzel, 2003 ▸, Grätzel, 2009 ▸; Chen *et al.*, 2012 ▸; Nguyen *et al.*, 2015 ▸). In order to fine tune its properties, great efforts have been made to develop new synthetic methods for function­alization of this bidentate ligand by introducing various substituents (Kaes *et al.*, 2000 ▸; Newkome *et al.*, 2004 ▸; Ortiz *et al.*, 2013 ▸; Norris *et al.*, 2013 ▸).

In this paper, we report on the synthesis of three 4-substituted 2,2′-bi­pyridine derivatives, namely 4-(4-methyl­phenyl­ethyn­yl)-2,2′-bi­pyridine, C_19_H_14_N_2_, (I), 4-(pyridin-3-ylethyn­yl)-2,2′-bi­pyridine, C_17_H_11_N_3_, (II) and 4-(indol-4-yl)-2,2′-bi­pyridine, C_18_H_13_N_3_, (III), obtained from the Sonogashira (Sonogashira *et al.*, 1975 ▸; Sonogashira, 2002 ▸; Negishi & de Meijere, 2002 ▸) and Suzuki–Miyaura (Miyaura & Suzuki, 1979 ▸; Suzuki, 1999 ▸; Kumar *et al.*, 2014 ▸; Blangetti *et al.*, 2013 ▸) cross-coupling reactions of 4-bromo-2,2′-bi­pyridine. The ethynyl bridge in (I) and (II) was introduced to decrease the steric hindrance between the pyridine ring and the aromatic substituent and at the same time to extend the π-conjugation. The crystal structures as well as geometry and the mol­ecular arrangement in the crystals of (I), (II) and (III) are reported herein.
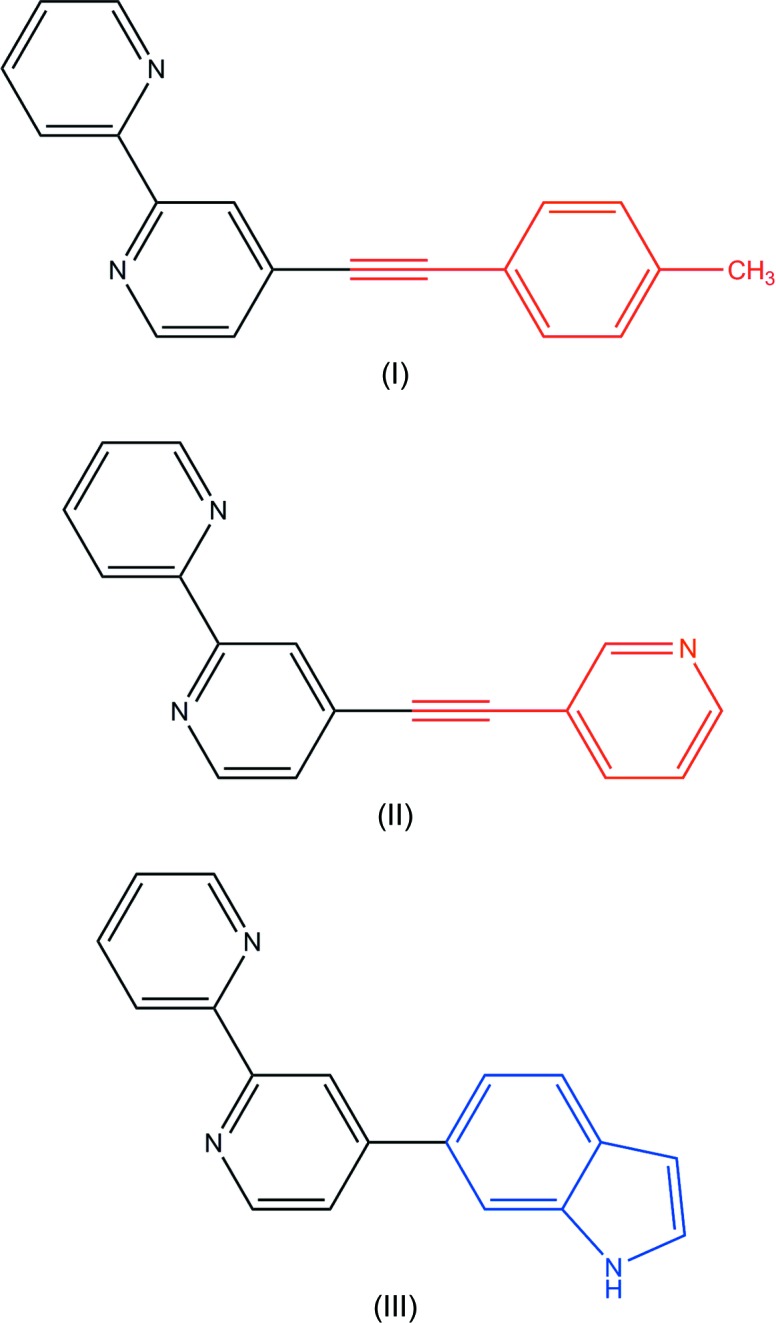


## Structural commentary   

The structures of the three 4-substituted 2,2′-bi­pyridines (I), (II), and (III) were elucidated by ^1^H and ^13^C NMR spectros­copy using d_1_-chloro­form as solvent (see *Synthesis and crystallization*). The ^1^H NMR spectra of the three compounds show typical proton resonances and splitting patterns of the Bpy core. The proton resonances of the introduced alkyne or the heteroarene moiety are easily recognized. In the ^13^C NMR spectrum of (I) and (II), the two resonance signals at about 94.3 and 86.5 p.p.m. prove the 2,2′-bi­pyridine and the tolyl or pyridine substituent to be connected by a C≡C linker. These signals typical for C*sp* carbons are not observed in the ^13^C NMR spectrum of (III) as the heterocycle is directly attached to the 2,2′-bi­pyridine core.

**Figure 1:**
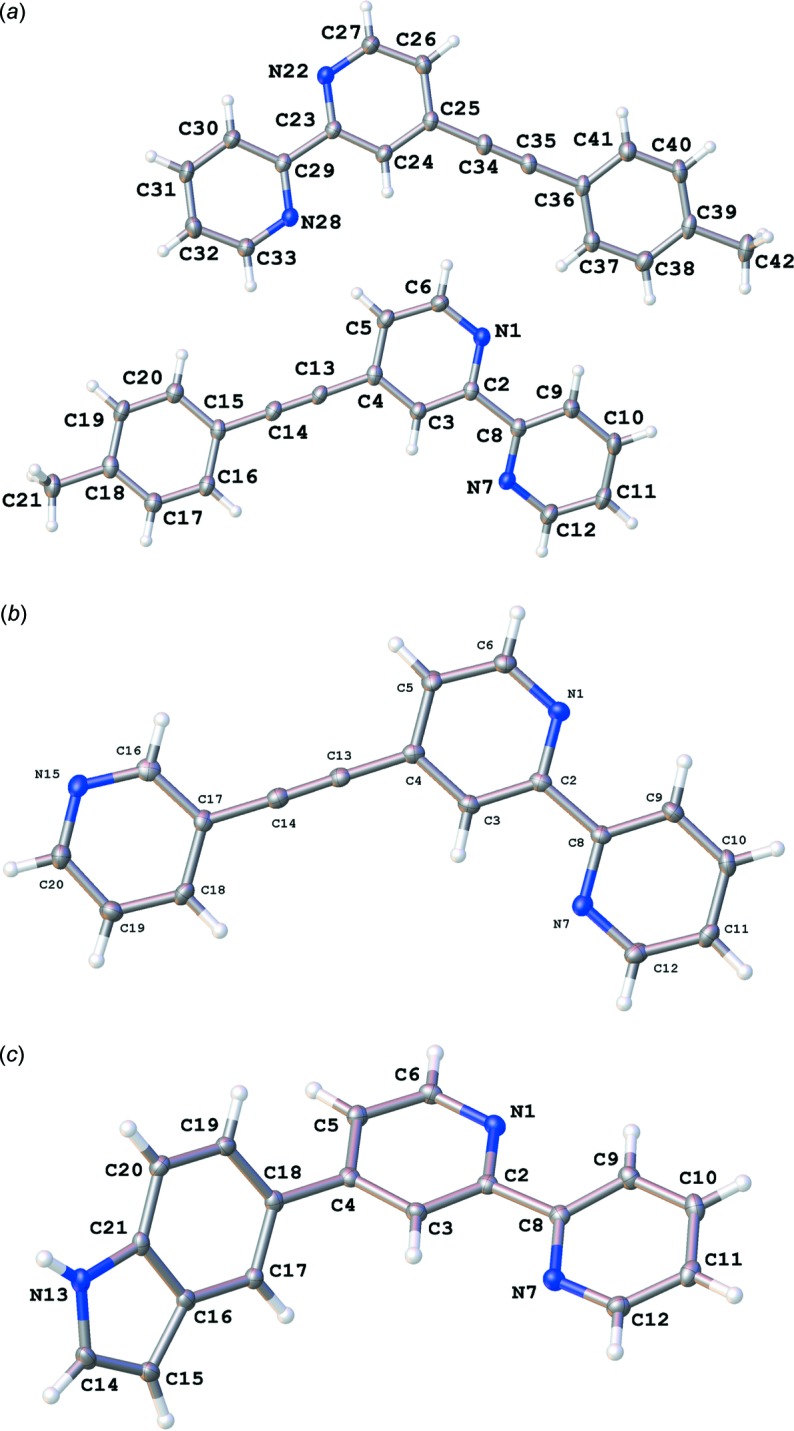
View of the asymmetric unit of (*a*) (I), (*b*) (II), and (*c*) (III) showing the atom-labelling schemes. Displacement ellipsoids are drawn at the 50% probability level.

The mol­ecular conformations of the compounds (I), (II) and (III) determined in the X-ray structural analysis are shown in Fig. 1 ▸. The asymmetric unit of (I) (Fig. 1 ▸ *a*) consists of two mol­ecules with similar conformational features (r.m.s deviation = 0.120 Å) and are linked by a C—H⋯N hydrogen bond (Table 1 ▸). As expected, the aromatic substituents introduced *via* an ethyl­ene bridge in (I) (Fig. 1 ▸ *a*) and (II) (Fig. 1 ▸ *b*) are essentially coplanar with the 2,2′-bi­pyridine core, as indicated by the dihedral angles between the aromatic moieties, *viz.* 8.98 (5) and 9.90 (6)° in (I) and 2.66 (14)° in (II). On the other hand, the indole moiety and the bipyridyl ring are out of plane in (III) (Fig. 1 ▸ *c*) in order to reduce the van de Waals repulsion between H5 with H19 and H3 with H17, the dihedral angle between the mean planes of the bi­pyridine core and indole ring being 55.82 (3)°.

The 2,2′-bipyridyl groups in the three compounds exhibit *trans* conformations and the pyridine rings are essentially co-planar, as indicated by the dihedral angles between the best planes through the two pyridine rings, *viz.* 3.40 (9) and 10.81 (9)° in (I), 0.4 (2)° in (II) and 11.66 (7)° in (III). These values are within the range 0.8–28.5° observed for the 2,2′-bi­pyridine derivatives substituted at the 4-position with an aromatic substituent (Table 4 ▸). All of these structural characteristics are consistent with those in our previous report (Nguyen *et al.*, 2014 ▸).

In conclusion, we have described facile synthetic procedures for 4-alkynylated and 4-aryl­ated 2,2′-bi­pyridines by means of the Sonogashira and Suzuki–Miyaura cross-coupling reactions of 4-bromo-2,2′-bi­pyridine. Based on this strategy, two novel 4-alkynylbi­pyridines and one 4-aryl-2,2′-bi­pyridine were synthesized whose structures were partially elucidated by NMR spectroscopic methods. In addition, the X-ray structural analysis revealed the planarity of the 4-alkynylbi­pyridines as the triple-bond linker separates the bi­pyridine and the introduced aromatic parts. This provides a hint for fine-tuning the electronic properties of this ligand by introducing suitable substituents. On the other hand, the introduced heterocyclic ring in compound (III), formed *via* Suzuki–Miyaura cross-coupling is twisted from the 2,2′-bi­pyridine ring due to the van der Waals repulsive force of the hydrogen atoms in close proximity.

## Supra­molecular features   

**Figure 2:**
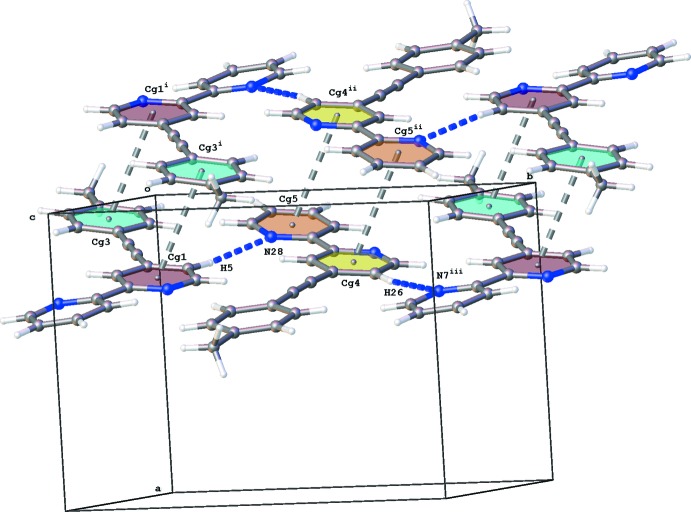
Partial crystal packing of (I) showing C—H⋯N (blue dotted lines) and π–π (gray dotted lines) inter­actions. [Symmetry codes: (i) −*x*, −*y*, −*z*; (ii) −*x*, −*y* + 1, −*z*; (iii) *x*, *y* + 1, *z*].

The crystal packing of (I) is dominated by π_pyridine_–π_pyridine_ and π_pyridine_–π_phen­yl_ stacking inter­actions [Fig. 2 ▸; *Cg*1⋯*Cg*3^i^ = 3.7769 (11) and *Cg*4⋯*Cg*5^ii^ = 3.8707 (11) Å; *Cg*1, *Cg*3, *Cg*4 and *Cg*5 are the centroids of the N1/C2–C6, C15–C20, N22/C23–27 and N28/C29–C33 rings, respectively; symmetry codes: (i) −*x*, −*y*, −*z*; (ii) −*x*, −*y* + 1, −*z*]. The mol­ecules lie in layers parallel to (30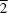) and within these planes, neighboring mol­ecules inter­act with each other through C—H⋯N hydrogen bonds (Table 1 ▸).

**Figure 3:**
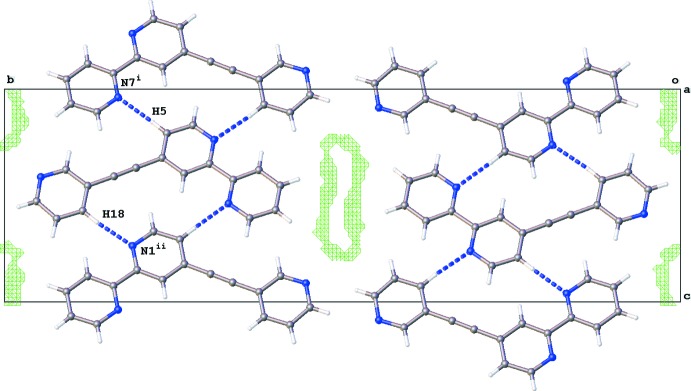
Crystal packing of (II) viewed along the *a* axis. C—H⋯N hydrogen bonds between neighboring columns of stacked mol­ecules are shown as blue dotted lines. Voids are contoured (green grid) at 0.2 Å away from the mol­ecular surface resulting in a total void volume of 375 Å^3^. [Symmetry codes: (i) *x* − 1, −*y* + 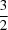, *z* − 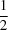; (ii) *x* + 1, −*y* + 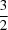, *z* + 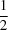].

Similarly, π–π inter­actions between the pyridine rings of (II) result in columms of mol­ecules along the *a*-axis direction [*Cg*1⋯*Cg*1^i^ = *Cg*2⋯*Cg*2^i^ = *Cg*3⋯*Cg*3^i^ = 3.7436 (3) Å; *Cg*1, *Cg*2, and *Cg*3 are centroids of the N1/C2–C6; N7/C7–C12 and N15/C16–C20 rings, respectively; symmetry code: (i) *x* + 1, *y*, *z*]. Neighboring columns inter­act by C—H⋯N hydrogen bonds (Fig. 3 ▸, Table 2 ▸). In between the columns, large voids (375 Å^3^) contain disordered solvent mol­ecules.

**Figure 4:**
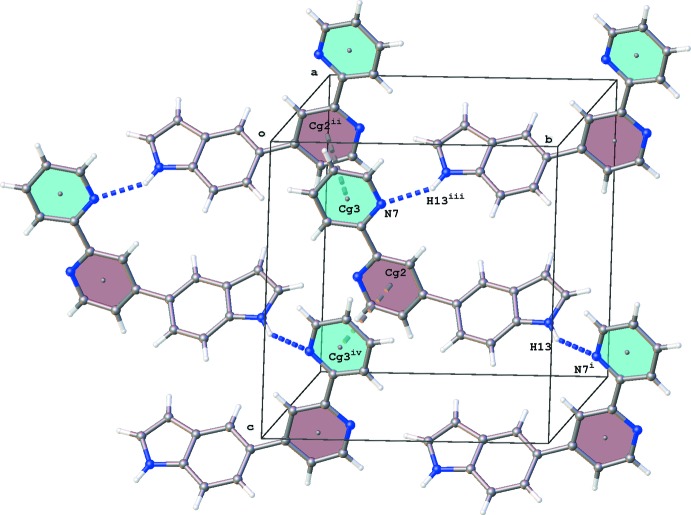
Crystal packing of (III) showing N—H⋯N hydrogen bonds (blue dotted lines) and π–π (gray dotted lines) inter­actions·[Symmetry codes: (i) *x*, −*y* + 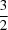, *z* + 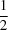; (ii) *x*, −*y* + 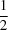, *z* − 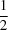; (iii) *x*, −*y* + 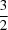, *z* − 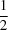; (iv) *x*, −*y* + 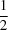, *z* + 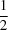].

The mol­ecules in the crystal packing of (III) are arranged in zigzag chains running along the *c* axis by hydrogen-bonding inter­actions in a head-to-tail manner between N13—H13⋯N7^i^ [symmetry code: (i) *x*, −*y* + 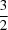, *z* + 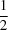; Table 3 ▸, Fig. 4 ▸]. These chains inter­act by π–π stacking between pyridine rings [*Cg*2⋯*Cg*3^i^ = 3.6920 (8) Å; *Cg*2 and *Cg*3 are the centroids of the N1/C2–C6 and N7/C8–C12 rings, respectively; symmetry code: (i) *x*, −*y* + 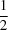, *z* + 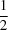] and C—H⋯π inter­actions (Table 3 ▸).

## Database survey   

An extension of the π-conjugated system of 2,2′-bi­pyridine can be obtained by the introduction of an aromatic substituent. A search in the Cambridge Structural Database (CSD, Version 5.38, last update February 2017; Groom *et al.*, 2016 ▸) for crystal structures of 2,2′-bi­pyridine derivatives substituted at the 4-position with an aromatic substituent resulted in 13 unique hits (excluding organometallic compounds) with substituents ranging from smaller phenyl and triazine rings to bi­pyridine, naphthalene, anthracene and phenanthrene to a larger pyrene ring (Table 4 ▸). However, it is evident from the dihedral angle between the best planes through pyridine and its aromatic 4-substituent (varying from 0.0 to 73.8°) that the degree of extension of the π-conjugated system depends on the steric hindrance of the substituent and the π–π inter­actions in the crystal packing.

## Synthesis and crystallization   

The compound 4-bromo-2,2′-bi­pyridine was prepared using literature procedures (Egbe *et al.*, 2001 ▸). The alkynylated and aryl­ated Bpy derivatives (I), (II), and (III) were prepared by the palladium-catalyzed Sonogashira and the palladium-catalyzed Suzuki–Miyaura cross-coupling reactions.

**(** ***a*** **) Synthesis of 4-(4-methyl­phenyl­ethyn­yl)-2,2′-bi­pyridine (I) by the Sonogashira reaction:** Toluene (4.0 ml) was deaerated by exchanging between a vacuum and a stream of argon (3 times). To this argon-saturated solution were added 4-bromo-2,2′-bi­pyridine (59 mg, 0.25 mmol, 1.0 equiv), Pd(PPh_3_)_4_ (28.5 mg, 0.025 mmol, 10 mol%) and CuI (10 mg, 0.050 mmol, 20 mol%). The pale-yellow mixture obtained was degassed again as described above. To the reaction mixture, a solution of *p*-tolyl­acetyl­ene (34.8 mg, 0.3 mmol, 1.2 equiv) in argon-saturated toluene (1.0 ml) was added dropwise over 15 minutes. The reaction mixture was heated at 323 K for 4 h. The reaction mixture turned reddish brown when the cross-coupling completed as indicated by TLC (EtOAc:*n*-hexane 1:4, *v*/*v*). The reaction mixture was diluted with EtOAc, washed with water (3 times), dried over anhydrous Na_2_SO_4_, and concentrated under reduced pressure. The residue was purified by SiO_2_ column chromatography to furnish the 4-alkynated 2,2′-bi­pyridine (I) as a brownish yellow solid (43 mg, 64%). M.p. 365–367 K; ^1^H NMR (CDCl_3_, 500 MHz): *δ* (p.p.m.) 8.70 (*dt*, *J* = 4.5 Hz and 0.5 Hz, 1 H), 8.65 (*d*, *J* = 5.0 Hz, 1 H), 8.52 (*s*, 1 H), 8.40 (*dd*, *J* = 8.0 Hz and 0.5 Hz, 1 H), 7.82 (*td*, *J* = 7.5 Hz and 1.5 Hz, 1 H), 7.45 (*d*, *J* = 8 Hz, 2 H, Ar), 7.38 (*dd*, *J* = 5.0 Hz and 1.0 Hz, 1 H), 7.32 (*m*, 1 H), 7.19 (*d*, *J* = 8 Hz, 2 H, Ar), 2.38 (*s*, 3 H, –CH_3_). ^13^C NMR (CDCl_3_, 125 MHz): *δ*(p.p.m.) 156.2, 155.6, 149.2, 149.1, 139.5, 137.0, 132.7, 131.8, 129.2, 125.2, 123.9, 123.2, 121.1, 119.2, 94.3 and 86.5 (C≡C), 21.6 (–CH_3_). Besides the desired cross-coupling product, a small amount of the Glaser homo-coupling by-product was also observed. Single crystals of (I) suitable for X-ray structure analysis were obtained by recrystallization from chloro­form.

**(** ***b*** **) 4-(Pyridine-3-ylethyn­yl)-2,2′-bi­pyridine (II):** Following the same procedure for (I), except that no CuI co-catalyst was used, (II) was obtained from 4-bromo-2,2′-bi­pyridine (59 mg, 0.25 mmol, 1.0 equiv) and pyridine-3-yl­acetyl­ene (31 mg, 0.3 mmol, 1.2 equiv) after 4 h at 373 K as a white solid (50 mg, 78%). M.p. 398–400 K; ^1^H NMR (CDCl_3_, 500 MHz): *δ* (p.p.m.) 8.81 (*s*, 1 H), 8.71 (*s*, 2 H), 8.62 (*dd*, *J* = 5.0 Hz and 1.0 Hz, 1 H), 8.57 (*s*, 1 H), 8.43 (*d*, *J* = 7.5 Hz, 1 H), 7.85 (*m*, 2 H), 7.42 (*d*, *J* = 8.0 Hz, 1 H), 7.33 (*m*, 2 H). ^13^C NMR (CDCl_3_, 125 MHz): *δ*(p.p.m.) 156.3, 155.3, 152.4, 149.4, 149.3, 149.2, 138.7, 137.0, 131.6, 125.1, 124.0, 123.2, 123.2, 121.2, 119.5, 90.2 (C≡C). Single crystals of (II) suitable for X-ray structure analysis were obtained by recrystallization from ethyl acetate.

**(** ***c*** **) Synthesis of 4-(1** ***H*** **-indol-4-yl)-2,2′-bi­pyridine (III) by the Suzuki–Miyaura reaction:** Toluene was degassed by exchanging between a vacuum and a stream of argon (3 times). 5-Bromo-2,2′-bi­pyridine (58 mg, 0.25 mmol, 1.0 equiv) and Pd(Ph_3_P)_4_ (28.8 mg, 0.025 mmol, 10 mol%) were dissolved in this degassed toluene (4 mL). To the obtained solution, H_2_O (1 ml), K_3_PO_4_ (105.5 mg, 0.5 mmol, 2.0 equiv), and 1*H*-indol-4-ylboronic acid (48.3 mg, 0.3 mmol, 1.2 equiv) were added. The reaction was stirred vigorously under an argon atmosphere at 383 K until TLC (*n*-hexa­ne–ethyl acetate 95:5,*v*/*v*) indicated the complete consumption of the starting material. The reaction mixture was filtered to remove insoluble particles. The filtrate was washed several times with H_2_O, dried over Na_2_SO_4_, and concentrated under reduced pressure by rotary evaporation. The residue was purified by SiO_2_ column chromatography (*n*-hexa­ne–ethyl acetate 97:3, *v*/*v*) to furnish the desired 4-aryl­ated 2,2′-bi­pyridine (III) as a yellow solid (32.5 mg, 48%). M.p. 356–357 K; ^1^H NMR (CDCl_3_, 500 MHz): *δ* (p.p.m.) 8.86 (*br s*, 1 H, NH indole), 8.74 (*m*, 2 H), 8.70 (*d*, *J* = 5.0 Hz, 1 H), 8.45 (*d*, *J* = 8.0 Hz, 1 H), 8.04 (*t*, *J* = 1.0 Hz, 1 H), 7.83 (*td*, *J* = 7.5 Hz and 2.0 Hz, 1 H), 7.60 (*dd*, *J* = 5.0 Hz and 2.0 Hz, 1 H), 7.55 (*dd*, *J* = 8.0 Hz and 2.0 Hz, 1 H), 7.42 (*d*, *J* = 7.5 Hz, 1 H), 7.31 (*m*, 1 H), 7.22 (*t*, *J* = 3.0 Hz, 1 H), 6.61 (*t*, *J* 2.0 Hz, 1 H). ^13^C NMR (CDCl_3_, 125 MHz)) : *δ*(p.p.m.) 156.5, 156.3, 150.7, 149.4, 149.1, 136.9, 136.4, 129.9, 128.5, 125.3, 123.7, 121.7, 121.4, 121.3, 119.6, 119.2, 111.6, 103.2. Single crystals of (III) suitable for X-ray structure analysis were obtained by recrystallization from chloro­form.

## Structure solution and refinement   

Crystal data, data collection and structure refinement details are summarized in Table 5 ▸. The structures of (I) and (III) were solved using *SHELXS97* (Sheldrick, 2008 ▸) and for (II) by charge flipping using *Olex2.solve* (Bourhis *et al.*, 2015 ▸). All hydrogen atoms were placed in idealized positions and refined in a riding mode with *U*_iso_(H) = 1.2 times those of their parent atoms (1.5 times for methyl groups), with C—H distances of 0.95 Å (aromatic) and 0.98 Å (CH_3_) and N—H distances of 0.88 Å.

For (II) a region of electron density amounting to the scattering from approximately 10.7 carbon atoms, apparently disordered in channels between columns of stacking mol­ecules, was removed with the SQUEEZE routine of *PLATON* (Spek, 2015 ▸) after it proved impossible to identify it with any reasonable solvent mol­ecule. A suggestion of possible twinning generated by *PLATON* (Spek, 2009 ▸) was further checked but subsequent refinement did not improve and was neglected.

## Supplementary Material

Crystal structure: contains datablock(s) global, II, III, I. DOI: 10.1107/S2056989017004662/zs2378sup1.cif

Structure factors: contains datablock(s) I. DOI: 10.1107/S2056989017004662/zs2378Isup2.hkl

Structure factors: contains datablock(s) II. DOI: 10.1107/S2056989017004662/zs2378IIsup3.hkl

Structure factors: contains datablock(s) III. DOI: 10.1107/S2056989017004662/zs2378IIIsup4.hkl

Supporting information file. DOI: 10.1107/S2056989017004662/zs2378Isup5.cml

Supporting information file. DOI: 10.1107/S2056989017004662/zs2378IIsup6.cml

Supporting information file. DOI: 10.1107/S2056989017004662/zs2378IIIsup7.cml

CCDC references: 1540011, 1540010, 1540009

Additional supporting information:  crystallographic information; 3D view; checkCIF report

## Figures and Tables

**Table 1:** Hydrogen-bond geometry (Å, °) for (I)

*D*—H⋯*A*	*D*—H	H⋯*A*	*D*⋯*A*	*D*—H⋯*A*
C5—H5⋯N28	0.95	2.53	3.472 (2)	169
C26—H26⋯N7^i^	0.95	2.55	3.487 (3)	171

Symmetry code: (i) 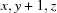.

**Table 2:** Hydrogen-bond geometry (Å, °) for (II)

*D*—H⋯*A*	*D*—H	H⋯*A*	*D*⋯*A*	*D*—H⋯*A*
C5—H5⋯N7^i^	0.95	2.55	3.475 (5)	163
C18—H18⋯N1^ii^	0.95	2.60	3.509 (5)	161

Symmetry codes: (i) 
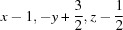
; (ii) 
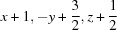
.

**Table 3:** Hydrogen-bond geometry (Å, °) for (III) *Cg*1, *Cg*2, *Cg*3 and *Cg*4 are the centroids of rings N13/C14–C16/C21, N1/C2–C6, N7/C8–C12 and C16–C21, respectively.

*D*—H⋯*A*	*D*—H	H⋯*A*	*D*⋯*A*	*D*—H⋯*A*
N13—H13⋯N7^i^	0.88	2.22	3.002 (2)	148
C14—H14⋯N1^ii^	0.95	2.39	3.336 (2)	176
C5—H5⋯*Cg*1^iii^	0.95	2.58	3.3371 (14)	137
C6—H6⋯*Cg*4^iii^	0.95	2.78	3.5268 (14)	136
C11—H11⋯*Cg*4^iv^	0.95	2.56	3.3548 (15)	141
C17—H17⋯*Cg*2^v^	0.95	2.85	3.6555 (15)	143
C20—H20⋯*Cg*3^vi^	0.95	2.86	3.5814 (16)	133

Symmetry codes: (i) 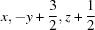; (ii) 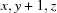; (iii) 
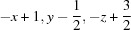
; (iv) 
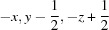
; (v) 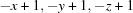; (vi) 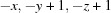.

**Table 4:** 4-Substituted 2,2′-bi­pyridines present in the Cambridge Structural Database^*a*^ The dihedral angle py–py is defined as the angle between the best planes through both pyridine rings and the dihedral angle py–Ar is defined as the angle between the best planes through the 4-substituted pyridine and the aromatic substituent.

4-Substituent	CSD refcode	Dihedral angle py–py (°)	Dihedral angle py–Ar (°)	Reference
(substituted) phen­yl	EWOYEW	0.8	9.1	Ramakrishnan *et al.* (2016 ▸)
	EWOXIZ	7.8/28.5/12.5	35.8/32.8/40.8	Ramakrishnan *et al.* (2016 ▸)
	ZOZRIF	6.6	24.5	Wang *et al.* (1996 ▸)
	RIPQUC	15.7	42.9	Cargill Thompson *et al.* (1997 ▸)
triazine	MULRUI	14.2/3.7/18.5	8.1/6.1/25.2	Laramée-Milette *et al.* (2015 ▸)
(substituted) naphthalene	EWOXUL	2.8/10.8/1.8	6.0/26.1/32.9	Ramakrishnan *et al.* (2016 ▸)
	EWOYIA	18.2/20.8	34.8/31.7	Ramakrishnan *et al.* (2016 ▸)
	OKAGOX	23.0/9.6	44.6/39.3	He *et al.* (2011 ▸)
2,2′-bi­pyridine	TEBGAI	3.2/2.7	0.0/0.0	Honey & Steel (1991 ▸)
anthracene	EWOWUK	4.0	73.8	Ramakrishnan *et al.* (2016 ▸)
phenanthrene	EWOXAR	5.2	64.8	Ramakrishnan *et al.* (2016 ▸)
	EWOXEV	11.1	53.1	Ramakrishnan *et al.* (2016 ▸)
pyrene	EWOXOF	4.0	51.6	Ramakrishnan *et al.* (2016 ▸)

Note: (*a*) Groom *et al.* (2016 ▸).

**Table 5:** Experimental details

	(I)	(II)	(III)
Crystal data			
Chemical formula	C_19_H_14_N_2_	C_17_H_11_N_3_	C_18_H_13_N_3_
*M*_r_	270.32	257.29	271.31
Crystal system, space group	Monoclinic, *P*2_1_/*c*	Monoclinic, *P*2_1_/*c*	Monoclinic, *P*2_1_/*c*
Temperature (K)	100	100	100
*a*, *b*, *c* (Å)	9.8697 (7), 12.6040 (7), 22.8414 (13)	3.7436 (3), 34.146 (3), 10.7528 (9)	9.6951 (6), 12.0142 (7), 12.0376 (9)
β (°)	97.890 (6)	94.799 (8)	109.552 (8)
*V* (Å^3^)	2814.5 (3)	1369.7 (2)	1321.28 (15)
*Z*	8	4	4
Radiation type	Mo *K*α	Mo *K*α	Mo *K*α
μ (mm^−1^)	0.08	0.08	0.08
Crystal size (mm)	0.30 × 0.15 × 0.10	0.40 × 0.10 × 0.10	0.35 × 0.35 × 0.20

Data collection			
Diffractometer	Agilent SuperNova (single source at offset, Eos detector)	Agilent SuperNova (single source at offset, Eos detector)	Agilent SuperNova (single source at offset, Eos detector)
Absorption correction	Multi-scan (*CrysAlis PRO*; Rigaku OD, 2015 ▸)	Multi-scan (*CrysAlis PRO*; Rigaku OD, 2015 ▸)	Multi-scan (*CrysAlis PRO*; Rigaku OD, 2015 ▸)
*T*_min_, *T*_max_	0.552, 1.000	0.695, 1.000	0.993, 1.000
No. of measured, independent and observed [*I* > 2σ(*I*)] reflections	12597, 5747, 3728	4235, 1926, 1645	8569, 2692, 2363
*R*_int_	0.025	0.022	0.023
θ_max_ (°)	26.4	23.3	26.4
(sin θ/λ)_max_ (Å^−1^)	0.625	0.555	0.625

Refinement			
*R*[*F* ^2^ > 2σ(*F* ^2^)], *wR*(*F* ^2^), *S*	0.054, 0.146, 1.04	0.083, 0.208, 1.15	0.038, 0.095, 1.06
No. of reflections	5747	1926	2692
No. of parameters	381	181	190
H-atom treatment	H-atom parameters constrained	H-atom parameters constrained	H-atom parameters constrained
Δρ_max_, Δρ_min_ (e Å^−3^)	0.24, −0.22	0.44, −0.29	0.21, −0.23

Computer programs: *CrysAlis PRO* (Rigaku OD, 2015 ▸), *SHELXS97* (Sheldrick, 2008 ▸), *Olex2.solve* (Bourhis *et al.*, 2015 ▸), *SHELXL2014* (Sheldrick, 2015 ▸) and *OLEX2* (Dolomanov *et al.*, 2009 ▸).

## supplementary crystallographic information

### (I) 4-[2-(4-Methylphenyl)ethynyl]-2,2'-bipyridine Crystal data

**Table:**

C_19_H_14_N_2_	*D*_x_ = 1.276 Mg m^−^^3^
*M**_r_* = 270.32	Melting point = 365–367 K
Monoclinic, *P*2_1_/*c*	Mo *K*α radiation, λ = 0.71073 Å
*a* = 9.8697 (7) Å	Cell parameters from 2955 reflections
*b* = 12.6040 (7) Å	θ = 3.0–28.1°
*c* = 22.8414 (13) Å	µ = 0.08 mm^−^^1^
β = 97.890 (6)°	*T* = 100 K
*V* = 2814.5 (3) Å^3^	Block, orange-colourless
*Z* = 8	0.30 × 0.15 × 0.10 mm
*F*(000) = 1136	

### (I) 4-[2-(4-Methylphenyl)ethynyl]-2,2'-bipyridine Data collection

**Table:**

Agilent SuperNova (single source at offset, Eos detector) diffractometer	5747 independent reflections
Radiation source: SuperNova (Mo) X-ray Source	3728 reflections with *I* > 2σ(*I*)
Mirror monochromator	*R*_int_ = 0.025
Detector resolution: 15.9631 pixels mm^-1^	θ_max_ = 26.4°, θ_min_ = 2.7°
ω scans	*h* = −12→9
Absorption correction: multi-scan (CrysAlis PRO; Rigaku OD, 2015)	*k* = −15→15
*T*_min_ = 0.552, *T*_max_ = 1.000	*l* = −25→28
12597 measured reflections	

### (I) 4-[2-(4-Methylphenyl)ethynyl]-2,2'-bipyridine Refinement

**Table:**

Refinement on *F*^2^	Primary atom site location: structure-invariant direct methods
Least-squares matrix: full	Secondary atom site location: difference Fourier map
*R*[*F*^2^ > 2σ(*F*^2^)] = 0.054	Hydrogen site location: inferred from neighbouring sites
*wR*(*F*^2^) = 0.146	H-atom parameters constrained
*S* = 1.04	*w* = 1/[σ^2^(*F*_o_^2^) + (0.0565*P*)^2^ + 0.7915*P*] where *P* = (*F*_o_^2^ + 2*F*_c_^2^)/3
5747 reflections	(Δ/σ)_max_ < 0.001
381 parameters	Δρ_max_ = 0.24 e Å^−^^3^
0 restraints	Δρ_min_ = −0.22 e Å^−^^3^

### (I) 4-[2-(4-Methylphenyl)ethynyl]-2,2'-bipyridine Special details

**Table:**

Geometry. All esds (except the esd in the dihedral angle between two l.s. planes) are estimated using the full covariance matrix. The cell esds are taken into account individually in the estimation of esds in distances, angles and torsion angles; correlations between esds in cell parameters are only used when they are defined by crystal symmetry. An approximate (isotropic) treatment of cell esds is used for estimating esds involving l.s. planes.
Refinement. Refinement of F^2^ against ALL reflections. The weighted R-factor wR and goodness of fit S are based on F^2^, conventional R-factors R are based on F, with F set to zero for negative F^2^. The threshold expression of F^2^ > 2sigma(F^2^) is used only for calculating R-factors(gt) etc. and is not relevant to the choice of reflections for refinement. R-factors based on F^2^ are statistically about twice as large as those based on F, and R- factors based on ALL data will be even larger.

### (I) 4-[2-(4-Methylphenyl)ethynyl]-2,2'-bipyridine Fractional atomic coordinates and isotropic or equivalent isotropic displacement parameters (Å^2^)

**Table:**

	*x*	*y*	*z*	*U*_iso_*/*U*_eq_	
C1D	0.44761 (19)	0.30825 (17)	0.43143 (8)	0.0253 (5)	
N22	0.21628 (16)	0.57648 (13)	0.03346 (7)	0.0249 (4)	
C23	0.20493 (18)	0.47210 (15)	0.04431 (8)	0.0208 (4)	
C24	0.23091 (18)	0.42918 (16)	0.10057 (8)	0.0219 (4)	
H24	0.2225	0.3549	0.1063	0.026*	
C25	0.26964 (18)	0.49598 (16)	0.14880 (8)	0.0219 (4)	
C26	0.28021 (19)	0.60404 (16)	0.13795 (9)	0.0258 (5)	
H26	0.3057	0.6525	0.1694	0.031*	
C27	0.2527 (2)	0.63910 (16)	0.08023 (9)	0.0277 (5)	
H27	0.2603	0.7130	0.0733	0.033*	
N28	0.15236 (16)	0.29935 (12)	0.00260 (7)	0.0233 (4)	
C29	0.16089 (18)	0.40394 (15)	−0.00772 (8)	0.0209 (4)	
C30	0.1289 (2)	0.44677 (16)	−0.06415 (8)	0.0265 (5)	
H30	0.1377	0.5208	−0.0705	0.032*	
C31	0.0845 (2)	0.38021 (16)	−0.11061 (9)	0.0300 (5)	
H31	0.0618	0.4079	−0.1494	0.036*	
C32	0.0731 (2)	0.27292 (17)	−0.10025 (9)	0.0281 (5)	
H32	0.0420	0.2252	−0.1314	0.034*	
C33	0.1085 (2)	0.23716 (16)	−0.04304 (9)	0.0252 (5)	
H33	0.1009	0.1633	−0.0359	0.030*	
C34	0.29879 (19)	0.45398 (16)	0.20791 (8)	0.0233 (5)	
C35	0.32897 (18)	0.41970 (16)	0.25663 (8)	0.0226 (4)	
C36	0.36776 (18)	0.38063 (16)	0.31578 (8)	0.0214 (4)	
C37	0.38186 (19)	0.27264 (16)	0.32746 (9)	0.0251 (5)	
H37	0.3647	0.2228	0.2961	0.030*	
C38	0.4208 (2)	0.23774 (16)	0.38463 (9)	0.0275 (5)	
H38	0.4294	0.1637	0.3921	0.033*	
C39	0.4325 (2)	0.41597 (17)	0.41955 (8)	0.0284 (5)	
H39	0.4490	0.4656	0.4510	0.034*	
C40	0.3939 (2)	0.45209 (17)	0.36263 (8)	0.0271 (5)	
H40	0.3851	0.5261	0.3553	0.033*	
C41	0.4909 (2)	0.26987 (19)	0.49369 (9)	0.0352 (5)	
H41A	0.5829	0.2963	0.5079	0.053*	
H41B	0.4913	0.1921	0.4942	0.053*	
H41C	0.4265	0.2962	0.5194	0.053*	
N1	0.30594 (16)	0.07848 (13)	0.21333 (7)	0.0237 (4)	
C2	0.29537 (18)	−0.02679 (15)	0.20395 (8)	0.0197 (4)	
C3	0.25839 (18)	−0.07017 (16)	0.14808 (8)	0.0209 (4)	
H3	0.2505	−0.1449	0.1432	0.025*	
C4	0.23300 (18)	−0.00324 (16)	0.09931 (8)	0.0212 (4)	
C5	0.24522 (18)	0.10603 (16)	0.10882 (8)	0.0240 (5)	
H5	0.2296	0.1547	0.0768	0.029*	
C6	0.28062 (19)	0.14124 (15)	0.16604 (8)	0.0245 (4)	
H6	0.2875	0.2157	0.1723	0.029*	
N7	0.34111 (16)	−0.19977 (13)	0.24599 (7)	0.0231 (4)	
C8	0.32952 (18)	−0.09575 (15)	0.25668 (8)	0.0202 (4)	
C9	0.3519 (2)	−0.05342 (16)	0.31331 (8)	0.0258 (5)	
H9	0.3407	0.0204	0.3195	0.031*	
C10	0.3908 (2)	−0.12057 (17)	0.36064 (8)	0.0285 (5)	
H10	0.4065	−0.0936	0.3998	0.034*	
C11	0.4063 (2)	−0.22683 (17)	0.34999 (9)	0.0290 (5)	
H11	0.4345	−0.2745	0.3815	0.035*	
C12	0.3800 (2)	−0.26281 (17)	0.29244 (9)	0.0284 (5)	
H12	0.3901	−0.3365	0.2854	0.034*	
C13	0.19552 (19)	−0.04592 (15)	0.04086 (8)	0.0216 (4)	
C14	0.16254 (19)	−0.08025 (16)	−0.00793 (8)	0.0231 (4)	
C15	0.12182 (18)	−0.12154 (16)	−0.06645 (8)	0.0220 (4)	
C16	0.10184 (19)	−0.22989 (16)	−0.07588 (9)	0.0243 (4)	
H16	0.1163	−0.2779	−0.0436	0.029*	
C17	0.06086 (19)	−0.26739 (16)	−0.13242 (9)	0.0250 (5)	
H17	0.0462	−0.3414	−0.1382	0.030*	
C18	0.04061 (19)	−0.20031 (16)	−0.18071 (8)	0.0253 (5)	
C19	0.06191 (19)	−0.09226 (16)	−0.17123 (8)	0.0265 (5)	
H19	0.0494	−0.0449	−0.2039	0.032*	
C20	0.10105 (19)	−0.05250 (16)	−0.11494 (8)	0.0250 (5)	
H20	0.1138	0.0217	−0.1092	0.030*	
C21	−0.0027 (2)	−0.24223 (17)	−0.24197 (9)	0.0319 (5)	
H21A	−0.0892	−0.2089	−0.2587	0.048*	
H21B	−0.0150	−0.3193	−0.2403	0.048*	
H21C	0.0678	−0.2258	−0.2669	0.048*	

### (I) 4-[2-(4-Methylphenyl)ethynyl]-2,2'-bipyridine Atomic displacement parameters (Å^2^)

**Table:**

	*U*^11^	*U*^22^	*U*^33^	*U*^12^	*U*^13^	*U*^23^
C1D	0.0183 (10)	0.0412 (13)	0.0158 (10)	−0.0012 (9)	0.0007 (8)	0.0047 (9)
N22	0.0308 (9)	0.0233 (9)	0.0196 (9)	−0.0029 (7)	0.0002 (7)	0.0013 (7)
C23	0.0205 (10)	0.0229 (11)	0.0185 (10)	0.0015 (8)	0.0005 (8)	0.0026 (8)
C24	0.0234 (10)	0.0223 (11)	0.0191 (10)	0.0009 (8)	−0.0001 (8)	0.0033 (8)
C25	0.0204 (10)	0.0272 (11)	0.0173 (10)	0.0007 (8)	0.0001 (8)	0.0015 (8)
C26	0.0295 (11)	0.0274 (11)	0.0195 (10)	−0.0028 (9)	−0.0006 (8)	−0.0028 (9)
C27	0.0366 (12)	0.0235 (11)	0.0220 (11)	−0.0024 (9)	0.0003 (9)	0.0027 (9)
N28	0.0273 (9)	0.0238 (9)	0.0184 (9)	0.0004 (7)	0.0014 (7)	0.0011 (7)
C29	0.0195 (10)	0.0248 (11)	0.0182 (10)	0.0022 (8)	0.0016 (8)	0.0025 (8)
C30	0.0332 (12)	0.0243 (11)	0.0206 (11)	−0.0001 (9)	−0.0017 (9)	0.0039 (8)
C31	0.0387 (12)	0.0337 (12)	0.0158 (10)	0.0006 (10)	−0.0026 (9)	0.0034 (9)
C32	0.0319 (11)	0.0318 (11)	0.0194 (11)	−0.0021 (9)	−0.0002 (9)	−0.0033 (9)
C33	0.0302 (11)	0.0238 (11)	0.0205 (11)	−0.0006 (9)	0.0002 (9)	0.0008 (8)
C34	0.0224 (10)	0.0288 (12)	0.0179 (11)	−0.0003 (8)	−0.0001 (8)	0.0001 (9)
C35	0.0209 (10)	0.0275 (11)	0.0191 (10)	0.0006 (8)	0.0012 (8)	−0.0017 (9)
C36	0.0181 (9)	0.0307 (11)	0.0150 (9)	0.0004 (8)	0.0011 (7)	0.0011 (8)
C37	0.0259 (10)	0.0305 (11)	0.0180 (11)	−0.0007 (9)	0.0003 (8)	−0.0006 (9)
C38	0.0297 (11)	0.0290 (12)	0.0229 (12)	−0.0009 (9)	0.0002 (9)	0.0041 (9)
C39	0.0308 (11)	0.0375 (13)	0.0165 (10)	0.0004 (9)	0.0019 (8)	−0.0050 (9)
C40	0.0299 (11)	0.0295 (12)	0.0214 (11)	0.0023 (9)	0.0014 (9)	−0.0004 (9)
C41	0.0327 (12)	0.0542 (15)	0.0180 (11)	−0.0016 (11)	0.0005 (9)	0.0055 (10)
N1	0.0274 (9)	0.0238 (9)	0.0190 (9)	−0.0005 (7)	−0.0003 (7)	0.0004 (7)
C2	0.0163 (9)	0.0252 (11)	0.0174 (10)	0.0006 (8)	0.0016 (7)	0.0010 (8)
C3	0.0217 (10)	0.0221 (11)	0.0185 (10)	0.0004 (8)	0.0013 (8)	0.0016 (8)
C4	0.0168 (9)	0.0291 (11)	0.0176 (10)	−0.0002 (8)	0.0027 (7)	0.0010 (8)
C5	0.0240 (10)	0.0261 (11)	0.0209 (10)	0.0001 (8)	−0.0009 (8)	0.0056 (8)
C6	0.0286 (11)	0.0199 (10)	0.0239 (11)	−0.0009 (8)	−0.0005 (8)	0.0031 (8)
N7	0.0272 (9)	0.0227 (9)	0.0189 (9)	0.0003 (7)	0.0012 (7)	0.0024 (7)
C8	0.0183 (9)	0.0246 (11)	0.0176 (10)	−0.0009 (8)	0.0016 (7)	0.0012 (8)
C9	0.0328 (11)	0.0258 (11)	0.0181 (10)	−0.0006 (9)	0.0015 (8)	−0.0016 (8)
C10	0.0360 (12)	0.0342 (12)	0.0147 (10)	−0.0028 (9)	0.0008 (9)	−0.0002 (9)
C11	0.0322 (12)	0.0363 (12)	0.0176 (11)	0.0021 (10)	0.0000 (9)	0.0070 (9)
C12	0.0352 (12)	0.0279 (12)	0.0215 (12)	0.0042 (9)	0.0013 (9)	0.0047 (9)
C13	0.0212 (10)	0.0244 (11)	0.0185 (10)	0.0003 (8)	0.0004 (8)	0.0054 (8)
C14	0.0221 (10)	0.0265 (11)	0.0203 (11)	0.0024 (8)	0.0016 (8)	0.0048 (9)
C15	0.0179 (9)	0.0314 (12)	0.0160 (10)	0.0006 (8)	0.0004 (8)	0.0003 (8)
C16	0.0245 (10)	0.0298 (11)	0.0179 (10)	0.0028 (9)	0.0007 (8)	0.0034 (9)
C17	0.0256 (11)	0.0276 (11)	0.0213 (11)	0.0009 (9)	0.0014 (8)	0.0010 (9)
C18	0.0203 (10)	0.0353 (12)	0.0196 (10)	0.0012 (9)	0.0006 (8)	−0.0003 (9)
C19	0.0266 (11)	0.0349 (12)	0.0175 (10)	−0.0002 (9)	0.0005 (8)	0.0069 (9)
C20	0.0254 (10)	0.0280 (11)	0.0214 (11)	0.0001 (8)	0.0025 (8)	0.0019 (8)
C21	0.0346 (12)	0.0403 (13)	0.0190 (12)	0.0007 (10)	−0.0025 (9)	0.0000 (9)

### (I) 4-[2-(4-Methylphenyl)ethynyl]-2,2'-bipyridine Geometric parameters (Å, º)

**Table:**

C1D—C38	1.387 (3)	N1—C2	1.346 (2)
C1D—C39	1.389 (3)	N1—C6	1.335 (2)
C1D—C41	1.507 (3)	C2—C3	1.390 (3)
N22—C23	1.346 (2)	C2—C8	1.486 (3)
N22—C27	1.337 (2)	C3—H3	0.9500
C23—C24	1.385 (3)	C3—C4	1.392 (3)
C23—C29	1.482 (3)	C4—C5	1.397 (3)
C24—H24	0.9500	C4—C13	1.440 (3)
C24—C25	1.398 (3)	C5—H5	0.9500
C25—C26	1.391 (3)	C5—C6	1.379 (3)
C25—C34	1.442 (3)	C6—H6	0.9500
C26—H26	0.9500	N7—C8	1.341 (2)
C26—C27	1.382 (3)	N7—C12	1.339 (2)
C27—H27	0.9500	C8—C9	1.389 (3)
N28—C29	1.344 (2)	C9—H9	0.9500
N28—C33	1.328 (2)	C9—C10	1.385 (3)
C29—C30	1.393 (3)	C10—H10	0.9500
C30—H30	0.9500	C10—C11	1.374 (3)
C30—C31	1.376 (3)	C11—H11	0.9500
C31—H31	0.9500	C11—C12	1.381 (3)
C31—C32	1.380 (3)	C12—H12	0.9500
C32—H32	0.9500	C13—C14	1.198 (3)
C32—C33	1.381 (3)	C14—C15	1.439 (3)
C33—H33	0.9500	C15—C16	1.392 (3)
C34—C35	1.193 (3)	C15—C20	1.401 (3)
C35—C36	1.440 (3)	C16—H16	0.9500
C36—C37	1.390 (3)	C16—C17	1.383 (3)
C36—C40	1.396 (3)	C17—H17	0.9500
C37—H37	0.9500	C17—C18	1.382 (3)
C37—C38	1.381 (3)	C18—C19	1.390 (3)
C38—H38	0.9500	C18—C21	1.502 (3)
C39—H39	0.9500	C19—H19	0.9500
C39—C40	1.381 (3)	C19—C20	1.385 (3)
C40—H40	0.9500	C20—H20	0.9500
C41—H41A	0.9800	C21—H21A	0.9800
C41—H41B	0.9800	C21—H21B	0.9800
C41—H41C	0.9800	C21—H21C	0.9800
			
C38—C1D—C39	118.15 (18)	C6—N1—C2	116.97 (16)
C38—C1D—C41	121.38 (19)	N1—C2—C3	122.60 (17)
C39—C1D—C41	120.47 (19)	N1—C2—C8	116.32 (16)
C27—N22—C23	116.74 (16)	C3—C2—C8	121.05 (18)
N22—C23—C24	122.87 (18)	C2—C3—H3	120.3
N22—C23—C29	116.20 (16)	C2—C3—C4	119.46 (19)
C24—C23—C29	120.93 (17)	C4—C3—H3	120.3
C23—C24—H24	120.3	C3—C4—C5	118.08 (18)
C23—C24—C25	119.46 (18)	C3—C4—C13	120.70 (18)
C25—C24—H24	120.3	C5—C4—C13	121.22 (17)
C24—C25—C34	120.98 (18)	C4—C5—H5	121.0
C26—C25—C24	117.94 (17)	C6—C5—C4	118.03 (18)
C26—C25—C34	121.07 (18)	C6—C5—H5	121.0
C25—C26—H26	120.9	N1—C6—C5	124.84 (19)
C27—C26—C25	118.26 (18)	N1—C6—H6	117.6
C27—C26—H26	120.9	C5—C6—H6	117.6
N22—C27—C26	124.72 (19)	C12—N7—C8	117.27 (17)
N22—C27—H27	117.6	N7—C8—C2	116.08 (16)
C26—C27—H27	117.6	N7—C8—C9	122.57 (17)
C33—N28—C29	117.57 (16)	C9—C8—C2	121.31 (18)
N28—C29—C23	116.51 (16)	C8—C9—H9	120.5
N28—C29—C30	122.05 (18)	C10—C9—C8	118.94 (19)
C30—C29—C23	121.43 (17)	C10—C9—H9	120.5
C29—C30—H30	120.5	C9—C10—H10	120.6
C31—C30—C29	119.01 (19)	C11—C10—C9	118.90 (18)
C31—C30—H30	120.5	C11—C10—H10	120.6
C30—C31—H31	120.3	C10—C11—H11	120.7
C30—C31—C32	119.32 (19)	C10—C11—C12	118.55 (19)
C32—C31—H31	120.3	C12—C11—H11	120.7
C31—C32—H32	121.1	N7—C12—C11	123.7 (2)
C31—C32—C33	117.79 (19)	N7—C12—H12	118.1
C33—C32—H32	121.1	C11—C12—H12	118.1
N28—C33—C32	124.24 (19)	C14—C13—C4	178.9 (2)
N28—C33—H33	117.9	C13—C14—C15	179.5 (2)
C32—C33—H33	117.9	C16—C15—C14	120.93 (18)
C35—C34—C25	177.1 (2)	C16—C15—C20	118.98 (18)
C34—C35—C36	178.5 (2)	C20—C15—C14	120.09 (18)
C37—C36—C35	121.45 (18)	C15—C16—H16	120.1
C37—C36—C40	118.79 (18)	C17—C16—C15	119.80 (18)
C40—C36—C35	119.75 (18)	C17—C16—H16	120.1
C36—C37—H37	120.0	C16—C17—H17	119.1
C38—C37—C36	120.05 (19)	C18—C17—C16	121.87 (19)
C38—C37—H37	120.0	C18—C17—H17	119.1
C1D—C38—H38	119.2	C17—C18—C19	118.20 (18)
C37—C38—C1D	121.55 (19)	C17—C18—C21	121.32 (19)
C37—C38—H38	119.2	C19—C18—C21	120.49 (18)
C1D—C39—H39	119.5	C18—C19—H19	119.5
C40—C39—C1D	120.98 (19)	C20—C19—C18	121.07 (18)
C40—C39—H39	119.5	C20—C19—H19	119.5
C36—C40—H40	119.8	C15—C20—H20	120.0
C39—C40—C36	120.49 (19)	C19—C20—C15	120.07 (19)
C39—C40—H40	119.8	C19—C20—H20	120.0
C1D—C41—H41A	109.5	C18—C21—H21A	109.5
C1D—C41—H41B	109.5	C18—C21—H21B	109.5
C1D—C41—H41C	109.5	C18—C21—H21C	109.5
H41A—C41—H41B	109.5	H21A—C21—H21B	109.5
H41A—C41—H41C	109.5	H21A—C21—H21C	109.5
H41B—C41—H41C	109.5	H21B—C21—H21C	109.5
			
C1D—C39—C40—C36	0.7 (3)	N1—C2—C3—C4	1.0 (3)
N22—C23—C24—C25	0.5 (3)	N1—C2—C8—N7	−169.28 (16)
N22—C23—C29—N28	178.53 (16)	N1—C2—C8—C9	8.8 (3)
N22—C23—C29—C30	−2.1 (3)	C2—N1—C6—C5	−0.2 (3)
C23—N22—C27—C26	0.6 (3)	C2—C3—C4—C5	−0.4 (3)
C23—C24—C25—C26	0.1 (3)	C2—C3—C4—C13	179.53 (17)
C23—C24—C25—C34	−179.39 (17)	C2—C8—C9—C10	−176.47 (17)
C23—C29—C30—C31	−177.98 (18)	C3—C2—C8—N7	9.0 (2)
C24—C23—C29—N28	−2.1 (3)	C3—C2—C8—C9	−172.87 (17)
C24—C23—C29—C30	177.27 (18)	C3—C4—C5—C6	−0.5 (3)
C24—C25—C26—C27	−0.4 (3)	C4—C5—C6—N1	0.9 (3)
C25—C26—C27—N22	0.0 (3)	C6—N1—C2—C3	−0.7 (3)
C27—N22—C23—C24	−0.9 (3)	C6—N1—C2—C8	177.53 (16)
C27—N22—C23—C29	178.43 (17)	N7—C8—C9—C10	1.5 (3)
N28—C29—C30—C31	1.4 (3)	C8—C2—C3—C4	−177.15 (16)
C29—C23—C24—C25	−178.73 (17)	C8—N7—C12—C11	1.0 (3)
C29—N28—C33—C32	1.0 (3)	C8—C9—C10—C11	0.2 (3)
C29—C30—C31—C32	−0.2 (3)	C9—C10—C11—C12	−1.1 (3)
C30—C31—C32—C33	−0.5 (3)	C10—C11—C12—N7	0.6 (3)
C31—C32—C33—N28	0.1 (3)	C12—N7—C8—C2	176.01 (16)
C33—N28—C29—C23	177.68 (16)	C12—N7—C8—C9	−2.1 (3)
C33—N28—C29—C30	−1.7 (3)	C13—C4—C5—C6	179.59 (17)
C34—C25—C26—C27	179.12 (18)	C14—C15—C16—C17	179.10 (17)
C35—C36—C37—C38	179.24 (18)	C14—C15—C20—C19	179.98 (17)
C35—C36—C40—C39	−179.36 (18)	C15—C16—C17—C18	0.9 (3)
C36—C37—C38—C1D	−0.5 (3)	C16—C15—C20—C19	−0.3 (3)
C37—C36—C40—C39	−0.3 (3)	C16—C17—C18—C19	−0.3 (3)
C38—C1D—C39—C40	−0.9 (3)	C16—C17—C18—C21	179.44 (18)
C39—C1D—C38—C37	0.8 (3)	C17—C18—C19—C20	−0.6 (3)
C40—C36—C37—C38	0.2 (3)	C18—C19—C20—C15	0.9 (3)
C41—C1D—C38—C37	−179.64 (18)	C20—C15—C16—C17	−0.6 (3)
C41—C1D—C39—C40	179.53 (18)	C21—C18—C19—C20	179.61 (18)

### (I) 4-[2-(4-Methylphenyl)ethynyl]-2,2'-bipyridine Hydrogen-bond geometry (Å, º)

**Table:**

*D*—H···*A*	*D*—H	H···*A*	*D*···*A*	*D*—H···*A*
C5—H5···N28	0.95	2.53	3.472 (2)	169
C26—H26···N7^i^	0.95	2.55	3.487 (3)	171

Symmetry code: (i) *x*, *y*+1, *z*.

### (II) 4-[2-(Pyridin-3-yl)ethynyl]-2,2'-bipyridine Crystal data

**Table:**

C_17_H_11_N_3_	*F*(000) = 536
*M**_r_* = 257.29	*D*_x_ = 1.248 Mg m^−^^3^
Monoclinic, *P*2_1_/*c*	Mo *K*α radiation, λ = 0.71073 Å
*a* = 3.7436 (3) Å	Cell parameters from 1697 reflections
*b* = 34.146 (3) Å	θ = 3.5–28.7°
*c* = 10.7528 (9) Å	µ = 0.08 mm^−^^1^
β = 94.799 (8)°	*T* = 100 K
*V* = 1369.7 (2) Å^3^	Needle, colourless
*Z* = 4	0.40 × 0.10 × 0.10 mm

### (II) 4-[2-(Pyridin-3-yl)ethynyl]-2,2'-bipyridine Data collection

**Table:**

Agilent SuperNova (single source at offset, Eos detector) diffractometer	1926 independent reflections
Radiation source: SuperNova (Mo) X-ray Source	1645 reflections with *I* > 2σ(*I*)
Detector resolution: 15.9631 pixels mm^-1^	*R*_int_ = 0.022
ω scans	θ_max_ = 23.3°, θ_min_ = 3.1°
Absorption correction: multi-scan (CrysAlis PRO; Rigaku OD, 2015)	*h* = −4→4
*T*_min_ = 0.695, *T*_max_ = 1.000	*k* = −37→33
4235 measured reflections	*l* = −11→11

### (II) 4-[2-(Pyridin-3-yl)ethynyl]-2,2'-bipyridine Refinement

**Table:**

Refinement on *F*^2^	0 restraints
Least-squares matrix: full	Hydrogen site location: inferred from neighbouring sites
*R*[*F*^2^ > 2σ(*F*^2^)] = 0.083	H-atom parameters constrained
*wR*(*F*^2^) = 0.208	*w* = 1/[σ^2^(*F*_o_^2^) + (0.0696*P*)^2^ + 4.7923*P*] where *P* = (*F*_o_^2^ + 2*F*_c_^2^)/3
*S* = 1.15	(Δ/σ)_max_ < 0.001
1926 reflections	Δρ_max_ = 0.44 e Å^−^^3^
181 parameters	Δρ_min_ = −0.29 e Å^−^^3^

### (II) 4-[2-(Pyridin-3-yl)ethynyl]-2,2'-bipyridine Special details

**Table:**

Geometry. All esds (except the esd in the dihedral angle between two l.s. planes) are estimated using the full covariance matrix. The cell esds are taken into account individually in the estimation of esds in distances, angles and torsion angles; correlations between esds in cell parameters are only used when they are defined by crystal symmetry. An approximate (isotropic) treatment of cell esds is used for estimating esds involving l.s. planes.

### (II) 4-[2-(Pyridin-3-yl)ethynyl]-2,2'-bipyridine Fractional atomic coordinates and isotropic or equivalent isotropic displacement parameters (Å^2^)

**Table:**

	*x*	*y*	*z*	*U*_iso_*/*U*_eq_	
N1	0.2656 (9)	0.68966 (10)	0.2378 (3)	0.0167 (8)	
C2	0.4386 (11)	0.69652 (11)	0.3508 (4)	0.0147 (9)	
C3	0.5026 (11)	0.73404 (11)	0.3962 (4)	0.0154 (9)	
H3	0.6208	0.7378	0.4770	0.018*	
C4	0.3928 (11)	0.76640 (12)	0.3231 (4)	0.0150 (9)	
C5	0.2126 (11)	0.75954 (12)	0.2070 (4)	0.0166 (10)	
H5	0.1294	0.7807	0.1548	0.020*	
C6	0.1571 (11)	0.72098 (12)	0.1693 (4)	0.0170 (10)	
H6	0.0343	0.7165	0.0897	0.020*	
N7	0.7312 (9)	0.66830 (10)	0.5380 (3)	0.0168 (8)	
C8	0.5578 (11)	0.66142 (11)	0.4254 (4)	0.0142 (9)	
C9	0.4899 (11)	0.62361 (12)	0.3803 (4)	0.0177 (10)	
H9	0.3668	0.6197	0.3004	0.021*	
C10	0.6034 (12)	0.59197 (12)	0.4530 (4)	0.0187 (10)	
H10	0.5582	0.5660	0.4244	0.022*	
C11	0.7850 (11)	0.59893 (12)	0.5686 (4)	0.0176 (10)	
H11	0.8691	0.5778	0.6206	0.021*	
C12	0.8406 (11)	0.63710 (12)	0.6063 (4)	0.0186 (10)	
H12	0.9646	0.6416	0.6857	0.022*	
C13	0.4658 (11)	0.80521 (12)	0.3695 (4)	0.0150 (9)	
C14	0.5307 (11)	0.83770 (12)	0.4099 (4)	0.0168 (10)	
N15	0.5621 (10)	0.94601 (10)	0.4131 (3)	0.0185 (9)	
C16	0.5068 (11)	0.90884 (12)	0.3788 (4)	0.0176 (10)	
H16	0.3938	0.9039	0.2979	0.021*	
C17	0.6047 (11)	0.87675 (11)	0.4544 (4)	0.0153 (9)	
C18	0.7784 (11)	0.88416 (12)	0.5721 (4)	0.0170 (10)	
H18	0.8565	0.8632	0.6258	0.020*	
C19	0.8344 (11)	0.92252 (12)	0.6090 (4)	0.0197 (10)	
H19	0.9457	0.9284	0.6895	0.024*	
C20	0.7262 (11)	0.95225 (12)	0.5273 (4)	0.0200 (10)	
H20	0.7701	0.9785	0.5533	0.024*	

### (II) 4-[2-(Pyridin-3-yl)ethynyl]-2,2'-bipyridine Atomic displacement parameters (Å^2^)

**Table:**

	*U*^11^	*U*^22^	*U*^33^	*U*^12^	*U*^13^	*U*^23^
N1	0.0152 (19)	0.0180 (19)	0.0163 (19)	−0.0010 (15)	−0.0013 (15)	−0.0006 (15)
C2	0.012 (2)	0.019 (2)	0.013 (2)	−0.0016 (18)	0.0030 (17)	−0.0006 (17)
C3	0.013 (2)	0.019 (2)	0.014 (2)	−0.0009 (18)	0.0014 (18)	−0.0006 (17)
C4	0.011 (2)	0.019 (2)	0.015 (2)	0.0016 (18)	0.0023 (17)	0.0005 (17)
C5	0.021 (2)	0.017 (2)	0.013 (2)	−0.0011 (18)	0.0048 (18)	0.0021 (16)
C6	0.017 (2)	0.021 (2)	0.013 (2)	0.0005 (18)	0.0021 (18)	−0.0003 (17)
N7	0.0190 (19)	0.0156 (19)	0.016 (2)	0.0019 (15)	0.0035 (15)	0.0008 (14)
C8	0.014 (2)	0.017 (2)	0.012 (2)	−0.0003 (17)	0.0029 (17)	−0.0003 (16)
C9	0.018 (2)	0.019 (2)	0.016 (2)	−0.0015 (18)	0.0005 (18)	−0.0038 (17)
C10	0.020 (2)	0.012 (2)	0.025 (2)	−0.0013 (18)	0.0066 (19)	−0.0031 (18)
C11	0.014 (2)	0.017 (2)	0.022 (2)	0.0028 (18)	0.0035 (19)	0.0048 (18)
C12	0.020 (2)	0.021 (2)	0.015 (2)	0.0047 (18)	0.0038 (18)	0.0017 (18)
C13	0.014 (2)	0.018 (2)	0.013 (2)	−0.0015 (18)	0.0024 (17)	0.0029 (18)
C14	0.014 (2)	0.020 (2)	0.016 (2)	0.0016 (18)	0.0020 (18)	0.0024 (18)
N15	0.024 (2)	0.0155 (18)	0.016 (2)	0.0005 (16)	0.0045 (16)	0.0019 (14)
C16	0.015 (2)	0.023 (2)	0.016 (2)	0.0004 (18)	0.0068 (18)	−0.0005 (18)
C17	0.016 (2)	0.015 (2)	0.016 (2)	0.0022 (18)	0.0057 (18)	−0.0006 (17)
C18	0.014 (2)	0.016 (2)	0.021 (2)	0.0029 (18)	0.0018 (18)	0.0047 (17)
C19	0.019 (2)	0.021 (2)	0.018 (2)	−0.0005 (19)	−0.0010 (19)	−0.0014 (18)
C20	0.021 (2)	0.015 (2)	0.024 (3)	0.0008 (18)	0.003 (2)	−0.0004 (18)

### (II) 4-[2-(Pyridin-3-yl)ethynyl]-2,2'-bipyridine Geometric parameters (Å, º)

**Table:**

N1—C2	1.348 (5)	C10—C11	1.387 (6)
N1—C6	1.342 (5)	C11—H11	0.9500
C2—C3	1.385 (6)	C11—C12	1.375 (6)
C2—C8	1.490 (6)	C12—H12	0.9500
C3—H3	0.9500	C13—C14	1.209 (6)
C3—C4	1.398 (6)	C14—C17	1.436 (6)
C4—C5	1.388 (6)	N15—C16	1.333 (5)
C4—C13	1.434 (6)	N15—C20	1.344 (6)
C5—H5	0.9500	C16—H16	0.9500
C5—C6	1.388 (6)	C16—C17	1.395 (6)
C6—H6	0.9500	C17—C18	1.397 (6)
N7—C8	1.345 (5)	C18—H18	0.9500
N7—C12	1.338 (5)	C18—C19	1.380 (6)
C8—C9	1.395 (6)	C19—H19	0.9500
C9—H9	0.9500	C19—C20	1.381 (6)
C9—C10	1.379 (6)	C20—H20	0.9500
C10—H10	0.9500		
			
C6—N1—C2	117.1 (3)	C11—C10—H10	120.7
N1—C2—C3	122.3 (4)	C10—C11—H11	120.7
N1—C2—C8	116.4 (3)	C12—C11—C10	118.5 (4)
C3—C2—C8	121.2 (4)	C12—C11—H11	120.7
C2—C3—H3	120.0	N7—C12—C11	124.1 (4)
C2—C3—C4	119.9 (4)	N7—C12—H12	117.9
C4—C3—H3	120.0	C11—C12—H12	117.9
C3—C4—C13	119.8 (4)	C14—C13—C4	179.1 (4)
C5—C4—C3	118.0 (4)	C13—C14—C17	178.4 (4)
C5—C4—C13	122.2 (4)	C16—N15—C20	116.9 (4)
C4—C5—H5	120.9	N15—C16—H16	118.0
C4—C5—C6	118.2 (4)	N15—C16—C17	124.0 (4)
C6—C5—H5	120.9	C17—C16—H16	118.0
N1—C6—C5	124.4 (4)	C16—C17—C14	120.1 (4)
N1—C6—H6	117.8	C16—C17—C18	117.8 (4)
C5—C6—H6	117.8	C18—C17—C14	122.2 (4)
C12—N7—C8	117.2 (3)	C17—C18—H18	120.7
N7—C8—C2	116.4 (3)	C19—C18—C17	118.7 (4)
N7—C8—C9	122.3 (4)	C19—C18—H18	120.7
C9—C8—C2	121.3 (4)	C18—C19—H19	120.5
C8—C9—H9	120.3	C18—C19—C20	119.1 (4)
C10—C9—C8	119.3 (4)	C20—C19—H19	120.5
C10—C9—H9	120.3	N15—C20—C19	123.5 (4)
C9—C10—H10	120.7	N15—C20—H20	118.2
C9—C10—C11	118.6 (4)	C19—C20—H20	118.2
			
N1—C2—C3—C4	−1.3 (6)	C8—N7—C12—C11	−0.4 (6)
N1—C2—C8—N7	179.9 (3)	C8—C9—C10—C11	−0.6 (6)
N1—C2—C8—C9	−0.5 (6)	C9—C10—C11—C12	0.7 (6)
C2—N1—C6—C5	0.2 (6)	C10—C11—C12—N7	−0.2 (7)
C2—C3—C4—C5	1.7 (6)	C12—N7—C8—C2	−179.9 (3)
C2—C3—C4—C13	−178.8 (4)	C12—N7—C8—C9	0.5 (6)
C2—C8—C9—C10	−179.6 (4)	C13—C4—C5—C6	179.3 (4)
C3—C2—C8—N7	−0.4 (6)	C14—C17—C18—C19	−178.9 (4)
C3—C2—C8—C9	179.3 (4)	N15—C16—C17—C14	179.3 (4)
C3—C4—C5—C6	−1.1 (6)	N15—C16—C17—C18	−1.5 (6)
C4—C5—C6—N1	0.2 (6)	C16—N15—C20—C19	−0.6 (6)
C6—N1—C2—C3	0.4 (6)	C16—C17—C18—C19	1.9 (6)
C6—N1—C2—C8	−179.9 (3)	C17—C18—C19—C20	−1.8 (6)
N7—C8—C9—C10	0.0 (6)	C18—C19—C20—N15	1.1 (7)
C8—C2—C3—C4	178.9 (4)	C20—N15—C16—C17	0.8 (6)

### (II) 4-[2-(Pyridin-3-yl)ethynyl]-2,2'-bipyridine Hydrogen-bond geometry (Å, º)

**Table:**

*D*—H···*A*	*D*—H	H···*A*	*D*···*A*	*D*—H···*A*
C5—H5···N7^i^	0.95	2.55	3.475 (5)	163
C18—H18···N1^ii^	0.95	2.60	3.509 (5)	161

Symmetry codes: (i) *x*−1, −*y*+3/2, *z*−1/2; (ii) *x*+1, −*y*+3/2, *z*+1/2.

### (III) 4-(Indol-4-yl)-2,2'-bipyridine Crystal data

**Table:**

C_18_H_13_N_3_	*D*_x_ = 1.364 Mg m^−^^3^
*M**_r_* = 271.31	Melting point = 398–400 K
Monoclinic, *P*2_1_/*c*	Mo *K*α radiation, λ = 0.71073 Å
*a* = 9.6951 (6) Å	Cell parameters from 4854 reflections
*b* = 12.0142 (7) Å	θ = 3.5–29.1°
*c* = 12.0376 (9) Å	µ = 0.08 mm^−^^1^
β = 109.552 (8)°	*T* = 100 K
*V* = 1321.28 (15) Å^3^	Block, orange
*Z* = 4	0.35 × 0.35 × 0.20 mm
*F*(000) = 568	

### (III) 4-(Indol-4-yl)-2,2'-bipyridine Data collection

**Table:**

Agilent SuperNova (single source at offset, Eos detector) diffractometer	2692 independent reflections
Radiation source: SuperNova (Mo) X-ray Source	2363 reflections with *I* > 2σ(*I*)
Mirror monochromator	*R*_int_ = 0.023
Detector resolution: 15.9631 pixels mm^-1^	θ_max_ = 26.4°, θ_min_ = 2.8°
ω scans	*h* = −12→12
Absorption correction: multi-scan (CrysAlis PRO; Rigaku OD, 2015)	*k* = −13→15
*T*_min_ = 0.993, *T*_max_ = 1.000	*l* = −15→11
8569 measured reflections	

### (III) 4-(Indol-4-yl)-2,2'-bipyridine Refinement

**Table:**

Refinement on *F*^2^	Primary atom site location: structure-invariant direct methods
Least-squares matrix: full	Secondary atom site location: difference Fourier map
*R*[*F*^2^ > 2σ(*F*^2^)] = 0.038	Hydrogen site location: inferred from neighbouring sites
*wR*(*F*^2^) = 0.095	H-atom parameters constrained
*S* = 1.06	*w* = 1/[σ^2^(*F*_o_^2^) + (0.0344*P*)^2^ + 0.7137*P*] where *P* = (*F*_o_^2^ + 2*F*_c_^2^)/3
2692 reflections	(Δ/σ)_max_ < 0.001
190 parameters	Δρ_max_ = 0.21 e Å^−^^3^
0 restraints	Δρ_min_ = −0.23 e Å^−^^3^

### (III) 4-(Indol-4-yl)-2,2'-bipyridine Special details

**Table:**

Geometry. All esds (except the esd in the dihedral angle between two l.s. planes) are estimated using the full covariance matrix. The cell esds are taken into account individually in the estimation of esds in distances, angles and torsion angles; correlations between esds in cell parameters are only used when they are defined by crystal symmetry. An approximate (isotropic) treatment of cell esds is used for estimating esds involving l.s. planes.
Refinement. Refinement of F^2^ against ALL reflections. The weighted R-factor wR and goodness of fit S are based on F^2^, conventional R-factors R are based on F, with F set to zero for negative F^2^. The threshold expression of F^2^ > 2sigma(F^2^) is used only for calculating R-factors(gt) etc. and is not relevant to the choice of reflections for refinement. R-factors based on F^2^ are statistically about twice as large as those based on F, and R- factors based on ALL data will be even larger.

### (III) 4-(Indol-4-yl)-2,2'-bipyridine Fractional atomic coordinates and isotropic or equivalent isotropic displacement parameters (Å^2^)

**Table:**

	*x*	*y*	*z*	*U*_iso_*/*U*_eq_	
N1	0.31593 (11)	0.25441 (9)	0.52186 (10)	0.0149 (2)	
C2	0.25581 (13)	0.33233 (11)	0.43923 (11)	0.0136 (3)	
C3	0.26375 (13)	0.44551 (11)	0.46516 (11)	0.0143 (3)	
H3	0.2241	0.4982	0.4039	0.017*	
C4	0.33001 (13)	0.48165 (11)	0.58118 (11)	0.0140 (3)	
C5	0.39151 (13)	0.40081 (11)	0.66627 (11)	0.0155 (3)	
H5	0.4381	0.4211	0.7464	0.019*	
C6	0.38351 (14)	0.29005 (11)	0.63189 (11)	0.0154 (3)	
H6	0.4290	0.2361	0.6904	0.018*	
N7	0.12564 (12)	0.36675 (9)	0.23212 (10)	0.0173 (3)	
C8	0.17196 (13)	0.29055 (11)	0.31882 (11)	0.0143 (3)	
C9	0.14054 (14)	0.17732 (11)	0.29891 (12)	0.0169 (3)	
H9	0.1737	0.1253	0.3617	0.020*	
C10	0.06031 (14)	0.14199 (11)	0.18635 (12)	0.0186 (3)	
H10	0.0385	0.0653	0.1709	0.022*	
C11	0.01227 (14)	0.21922 (12)	0.09662 (12)	0.0183 (3)	
H11	−0.0428	0.1971	0.0186	0.022*	
C12	0.04713 (14)	0.33039 (12)	0.12420 (12)	0.0181 (3)	
H12	0.0133	0.3838	0.0628	0.022*	
N13	0.25953 (12)	0.93647 (9)	0.65298 (10)	0.0172 (3)	
H13	0.2204	0.9745	0.6974	0.021*	
C14	0.31571 (14)	0.98129 (11)	0.57170 (12)	0.0182 (3)	
H14	0.3184	1.0585	0.5553	0.022*	
C15	0.36687 (14)	0.89859 (11)	0.51831 (12)	0.0170 (3)	
H15	0.4112	0.9076	0.4594	0.020*	
C16	0.34102 (13)	0.79562 (11)	0.56791 (11)	0.0143 (3)	
C17	0.36292 (13)	0.68330 (11)	0.54613 (11)	0.0142 (3)	
H17	0.4045	0.6637	0.4877	0.017*	
C18	0.32337 (13)	0.60125 (11)	0.61069 (11)	0.0139 (3)	
C19	0.26479 (14)	0.63113 (11)	0.69974 (11)	0.0149 (3)	
H19	0.2429	0.5742	0.7460	0.018*	
C20	0.23857 (14)	0.74072 (11)	0.72121 (11)	0.0156 (3)	
H20	0.1974	0.7597	0.7801	0.019*	
C21	0.27468 (13)	0.82248 (11)	0.65331 (11)	0.0146 (3)	

### (III) 4-(Indol-4-yl)-2,2'-bipyridine Atomic displacement parameters (Å^2^)

**Table:**

	*U*^11^	*U*^22^	*U*^33^	*U*^12^	*U*^13^	*U*^23^
N1	0.0145 (5)	0.0133 (5)	0.0168 (6)	0.0003 (4)	0.0051 (4)	0.0009 (4)
C2	0.0121 (6)	0.0143 (6)	0.0149 (6)	0.0002 (5)	0.0054 (5)	0.0004 (5)
C3	0.0150 (6)	0.0130 (6)	0.0143 (6)	0.0006 (5)	0.0040 (5)	0.0019 (5)
C4	0.0121 (6)	0.0140 (6)	0.0169 (7)	−0.0011 (5)	0.0061 (5)	0.0003 (5)
C5	0.0132 (6)	0.0176 (7)	0.0143 (6)	−0.0012 (5)	0.0026 (5)	−0.0004 (5)
C6	0.0138 (6)	0.0148 (6)	0.0167 (6)	0.0018 (5)	0.0041 (5)	0.0035 (5)
N7	0.0182 (5)	0.0159 (6)	0.0166 (6)	−0.0010 (4)	0.0045 (4)	0.0009 (5)
C8	0.0132 (6)	0.0148 (6)	0.0158 (6)	0.0007 (5)	0.0060 (5)	−0.0003 (5)
C9	0.0165 (6)	0.0142 (7)	0.0193 (7)	0.0014 (5)	0.0050 (5)	0.0006 (5)
C10	0.0146 (6)	0.0155 (7)	0.0244 (7)	−0.0004 (5)	0.0050 (5)	−0.0052 (6)
C11	0.0133 (6)	0.0244 (7)	0.0165 (7)	−0.0009 (5)	0.0039 (5)	−0.0053 (6)
C12	0.0183 (6)	0.0196 (7)	0.0156 (7)	0.0005 (5)	0.0044 (5)	0.0018 (5)
N13	0.0210 (6)	0.0122 (6)	0.0171 (6)	0.0021 (5)	0.0049 (4)	−0.0019 (4)
C14	0.0223 (7)	0.0124 (6)	0.0164 (7)	−0.0016 (5)	0.0017 (5)	0.0015 (5)
C15	0.0208 (6)	0.0147 (6)	0.0142 (6)	−0.0029 (5)	0.0040 (5)	0.0010 (5)
C16	0.0134 (6)	0.0151 (6)	0.0118 (6)	−0.0013 (5)	0.0007 (5)	−0.0005 (5)
C17	0.0137 (6)	0.0145 (6)	0.0133 (6)	−0.0003 (5)	0.0031 (5)	−0.0015 (5)
C18	0.0118 (6)	0.0143 (6)	0.0130 (6)	−0.0001 (5)	0.0009 (5)	−0.0009 (5)
C19	0.0161 (6)	0.0149 (6)	0.0128 (6)	−0.0024 (5)	0.0036 (5)	0.0007 (5)
C20	0.0153 (6)	0.0184 (7)	0.0133 (6)	−0.0003 (5)	0.0050 (5)	−0.0018 (5)
C21	0.0140 (6)	0.0128 (6)	0.0141 (6)	0.0003 (5)	0.0006 (5)	−0.0025 (5)

### (III) 4-(Indol-4-yl)-2,2'-bipyridine Geometric parameters (Å, º)

**Table:**

N1—C2	1.3481 (17)	C11—C12	1.3904 (19)
N1—C6	1.3365 (17)	C12—H12	0.9500
C2—C3	1.3915 (18)	N13—H13	0.8800
C2—C8	1.4916 (18)	N13—C14	1.3786 (18)
C3—H3	0.9500	N13—C21	1.3773 (17)
C3—C4	1.3969 (18)	C14—H14	0.9500
C4—C5	1.3925 (18)	C14—C15	1.3637 (19)
C4—C18	1.4867 (18)	C15—H15	0.9500
C5—H5	0.9500	C15—C16	1.4318 (18)
C5—C6	1.3880 (18)	C16—C17	1.4043 (18)
C6—H6	0.9500	C16—C21	1.4202 (18)
N7—C8	1.3473 (17)	C17—H17	0.9500
N7—C12	1.3401 (17)	C17—C18	1.3864 (18)
C8—C9	1.3972 (18)	C18—C19	1.4170 (18)
C9—H9	0.9500	C19—H19	0.9500
C9—C10	1.3841 (19)	C19—C20	1.3817 (18)
C10—H10	0.9500	C20—H20	0.9500
C10—C11	1.381 (2)	C20—C21	1.3953 (19)
C11—H11	0.9500		
			
C6—N1—C2	117.14 (11)	N7—C12—H12	118.0
N1—C2—C3	122.36 (12)	C11—C12—H12	118.0
N1—C2—C8	116.34 (11)	C14—N13—H13	125.6
C3—C2—C8	121.20 (11)	C21—N13—H13	125.6
C2—C3—H3	120.0	C21—N13—C14	108.87 (11)
C2—C3—C4	120.03 (12)	N13—C14—H14	125.0
C4—C3—H3	120.0	C15—C14—N13	110.02 (12)
C3—C4—C18	119.81 (11)	C15—C14—H14	125.0
C5—C4—C3	117.33 (12)	C14—C15—H15	126.5
C5—C4—C18	122.72 (12)	C14—C15—C16	106.91 (12)
C4—C5—H5	120.6	C16—C15—H15	126.5
C6—C5—C4	118.81 (12)	C17—C16—C15	133.94 (12)
C6—C5—H5	120.6	C17—C16—C21	119.13 (12)
N1—C6—C5	124.24 (12)	C21—C16—C15	106.88 (11)
N1—C6—H6	117.9	C16—C17—H17	120.3
C5—C6—H6	117.9	C18—C17—C16	119.41 (12)
C12—N7—C8	117.60 (12)	C18—C17—H17	120.3
N7—C8—C2	117.13 (11)	C17—C18—C4	120.74 (12)
N7—C8—C9	122.12 (12)	C17—C18—C19	120.00 (12)
C9—C8—C2	120.73 (12)	C19—C18—C4	119.07 (11)
C8—C9—H9	120.5	C18—C19—H19	119.1
C10—C9—C8	119.03 (13)	C20—C19—C18	121.89 (12)
C10—C9—H9	120.5	C20—C19—H19	119.1
C9—C10—H10	120.3	C19—C20—H20	121.2
C11—C10—C9	119.44 (13)	C19—C20—C21	117.60 (12)
C11—C10—H10	120.3	C21—C20—H20	121.2
C10—C11—H11	121.1	N13—C21—C16	107.31 (11)
C10—C11—C12	117.85 (12)	N13—C21—C20	130.87 (12)
C12—C11—H11	121.1	C20—C21—C16	121.82 (12)
N7—C12—C11	123.95 (13)		
			
N1—C2—C3—C4	−2.97 (19)	C10—C11—C12—N7	0.7 (2)
N1—C2—C8—N7	−172.50 (11)	C12—N7—C8—C2	−178.40 (11)
N1—C2—C8—C9	9.08 (17)	C12—N7—C8—C9	0.00 (19)
C2—N1—C6—C5	2.00 (19)	N13—C14—C15—C16	0.34 (15)
C2—C3—C4—C5	2.62 (18)	C14—N13—C21—C16	−0.78 (14)
C2—C3—C4—C18	−173.06 (11)	C14—N13—C21—C20	178.21 (13)
C2—C8—C9—C10	178.90 (12)	C14—C15—C16—C17	176.75 (14)
C3—C2—C8—N7	10.94 (18)	C14—C15—C16—C21	−0.80 (14)
C3—C2—C8—C9	−167.48 (12)	C15—C16—C17—C18	−179.18 (13)
C3—C4—C5—C6	−0.18 (18)	C15—C16—C21—N13	0.97 (14)
C3—C4—C18—C17	−50.68 (17)	C15—C16—C21—C20	−178.13 (11)
C3—C4—C18—C19	124.26 (13)	C16—C17—C18—C4	173.34 (11)
C4—C5—C6—N1	−2.2 (2)	C16—C17—C18—C19	−1.55 (18)
C4—C18—C19—C20	−171.75 (11)	C17—C16—C21—N13	−177.02 (11)
C5—C4—C18—C17	133.88 (13)	C17—C16—C21—C20	3.88 (18)
C5—C4—C18—C19	−51.18 (17)	C17—C18—C19—C20	3.23 (19)
C6—N1—C2—C3	0.64 (18)	C18—C4—C5—C6	175.36 (11)
C6—N1—C2—C8	−175.87 (11)	C18—C19—C20—C21	−1.27 (19)
N7—C8—C9—C10	0.56 (19)	C19—C20—C21—N13	178.86 (13)
C8—C2—C3—C4	173.38 (11)	C19—C20—C21—C16	−2.28 (18)
C8—N7—C12—C11	−0.6 (2)	C21—N13—C14—C15	0.28 (15)
C8—C9—C10—C11	−0.48 (19)	C21—C16—C17—C18	−1.86 (18)
C9—C10—C11—C12	−0.12 (19)		

### (III) 4-(Indol-4-yl)-2,2'-bipyridine Hydrogen-bond geometry (Å, º)

Cg1, Cg2, Cg3 and Cg4 are the centroids of rings N13/C14–C16/C21, N1/C2–C6, N7/C8–C12 and C16–C21, respectively.

**Table:**

*D*—H···*A*	*D*—H	H···*A*	*D*···*A*	*D*—H···*A*
N13—H13···N7^i^	0.88	2.22	3.002 (2)	148
C14—H14···N1^ii^	0.95	2.39	3.336 (2)	176
C5—H5···*Cg*1^iii^	0.95	2.58	3.3371 (14)	137
C6—H6···*Cg*4^iii^	0.95	2.78	3.5268 (14)	136
C11—H11···*Cg*4^iv^	0.95	2.56	3.3548 (15)	141
C17—H17···*Cg*2^v^	0.95	2.85	3.6555 (15)	143
C20—H20···*Cg*3^vi^	0.95	2.86	3.5814 (16)	133

Symmetry codes: (i) *x*, −*y*+3/2, *z*+1/2; (ii) *x*, *y*+1, *z*; (iii) −*x*+1, *y*−1/2, −*z*+3/2; (iv) −*x*, *y*−1/2, −*z*+1/2; (v) −*x*+1, −*y*+1, −*z*+1; (vi) −*x*, −*y*+1, −*z*+1.
